# High-risk molecular features may eclipse genomic complexity in predicting chronic lymphocytic leukemia outcomes; UK clinical trial insights

**DOI:** 10.1038/s41375-026-02906-5

**Published:** 2026-03-11

**Authors:** H. Parker, L. Carr, K. Norris, A. Nilsson-Takeuchi, B. Stevens, H. Amarasinghe, L. Kadalayil, M. Else, R. Clifford, A. Pettitt, T. Munir, A. Schuh, R. Walewska, D. M. Baird, D. G. Oscier, C. Pepper, D. Bryant, J. Gibson, JC Strefford

**Affiliations:** 1 Cancer Genomics, School of Cancer Sciences, University of Southampton, Southampton, UK; 2 Division of Cancer and Genetics, School of Medicine, Cardiff University, Cardiff, UK; 3 School of Chemistry, Faculty of Engineering and Physical Sciences, University of Southampton, Southampton, UK; 4 Division of Cancer Biology, The Institute of Cancer Research, London, UK; 5 University of Oxford, Oxford, UK; 6 University of Liverpool, Liverpool, UK; 7 Leeds Cancer Centre, Leeds Teaching Hospitals NHS Trust, Leeds, UK; 8 Oxford University Hospitals NHS Foundation Trust, Oxford, UK; 9 Division of Haematology, University Hospitals Dorset, Bournemouth, UK; 10 Brighton and Sussex Medical School, University of Sussex, Brighton, UK

**Keywords:** Chronic lymphocytic leukaemia, Cancer genomics

## Abstract

High genomic complexity (HGC) is linked to poor prognosis in CLL, but its independent prognostic value remains uncertain amid emerging biomarkers. We analysed copy number alterations (CNA) in 495 untreated patients from (immuno)chemotherapy trials (CLL4, ADMIRE, ARCTIC), incorporating IGHV status, telomere length (TL), targeted sequencing and DNA-methylation subtypes. Patients harboured low (LGC, ≤2 CNAs; *n* = 334), intermediate (IGC, 3–4 CNAs; *n* = 97), or high (HGC, ≥5 CNAs; *n* = 64) genomic complexity. HGC associated with U-CLL (81%, *p* < 0.001), *TP53*-aberration (36%, *p* < 0.001), short TL (TL-S; 61%, *p* < 0.05), del13q (50%, *p* < 0.001) and del11q (22%, *p* < 0.05). IGC was enriched for biallelic *ATM* disruption and *BIRC3* deletions (*p* < 0.001). Trisomy 12 and *NOTCH1* mutations were enriched in LGC (*p* < 0.001). HGC associated with shorter progression-free and overall survival in univariate models but only remained independent for OS in CLL4 (HR = 1.61, *p* = 0.02). Independent prognostic factors included *TP53* aberration, U-CLL, TL-S and n-CLL. Of 64 HGC patients, 23 had *TP53*-aberration; 92% of *TP53* wild-type cases had other high-risk features (TL-S, U-CLL, or n-CLL). HGC may reflect a convergence of high-risk features rather than represent an independent biomarker. The interplay of telomere attrition, IGHV status and DNA methylation subtype necessitates further validation in targeted therapy cohorts to enhance risk assessment in prognostic models.

## Introduction

Chronic lymphocytic leukaemia (CLL) is a heterogeneous disease characterised by significant biological and clinical variability, affecting disease progression, treatment response and patient outcomes. Key prognostic indicators, such as *TP53* aberrations and unmutated IGHV genes (U-CLL), are essential for risk stratification but do not include all patients at risk of poor survival. As CLL cells proliferate in the bone marrow and lymph nodes, some clones develop complex genomes marked by structural lesions, aneuploidy and somatic mutations. Genomic complexity (GC) is defined by chromosomal alterations: high-GC (HGC) is defined by five or more karyotypic or copy number alterations (CNAs), while GC encompasses three or more. Notably, although *TP53* aberrations precede the evolution of GC [1], over 20% of GC cases lack detectable *TP53* lesions, indicating that other factors may contribute to GC and certain chromosomal changes like trisomy 12 and trisomy 19 may positively or neutrally impact clinical outcomes [2]. Sequential chromosomal analysis frequently shows clonal evolution, especially in cases with an abnormal karyotype at presentation. Cases that progress after initial therapy and show further chromosomal evolution have a poorer response to second-line therapies than those with stable karyotypes [3].

Recent studies have linked HGC to poorer prognosis [4–7] and an increased risk of Richter’s transformation [8], often associating with negative indicators like *TP53* aberration and unmutated IGHV CLL (U-CLL) [9, 10]. In the context of traditional chemotherapy, chlorambucil-based regimens showed improved overall survival (OS) for patients without GC compared to those with HGC [11]. The CLL14 trial indicated that HGC patients had lower response rates and significantly shorter progression-free survival (PFS) and OS compared to patients without GC [12]. For targeted treatments, while some data suggest shorter OS for HGC patients treated with ibrutinib [13], other studies reported high overall response rates, though with shorter durations and poorer PFS [14]. The MURANO study revealed that HGC patients on venetoclax had lower rates of undetectable measurable residual disease (uMRD) and shorter PFS [15], which the CLL13 trial confirmed [16]. Interestingly, the CLL14 trial found no significant differences in response rates or survival between HGC and non-GC patients [12]. Similarly, treatment with the PI3K-delta inhibitor, idelalisib, showed no significant difference in response rates for complex karyotype (CK) and non-CK patients [17].

Additionally, HGC often coexists with telomere length (TL) attrition [18]. Critical TL shortening leads to uncapped telomeres, driving fusion events and accumulating genetic lesions like del(17p) and del(11q), thus contributing to HGC [18–21]. CLL cells demonstrate telomere disruption-induced foci (TIF) [22], marked by gamma H2AX and 53BP1 localisation and exhibit telomere deletions and terminal duplications [23] as well as telomere fusion events [19]. Short TL correlates with poor prognostic deletions [17p, 11q] and inferior survival [24–26]. The relationship between TL and IGHV mutation status is established, with shorter telomeres in U-CLL cases and a significant positive correlation between TL and mutational load at the IGHV locus [19, 27, 28]. TL in mutated IGHV CLLs (M-CLL) is more heterogeneous, perhaps the result of variable numbers of cell division in response to antigen-mediated germinal centre reactions, where telomerase is activated to maintain TL [28–30], The importance of the combination of proliferative potential and GC is further exemplified by CLL cases with mutated IGHV genes (M-CLL) that have *TP53* aberrations and karyotypic complexity, yet indolent disease [31]. Analysis of the CLL methylome identified three clinically relevant epitypes linked to CpG modifications in naive (n-CLL) and memory (m-CLL) B cells and a third epitype with intermediate (i-CLL) methylation and moderate IGHV mutations, particularly enriched for IGLV3–21 R110 and *SF3B1* mutations [32–34]. These epitypes correlate with clinical outcomes [35] and differ in frequencies of *TP53* aberrations and TL [36].

Given that measuring GC is not trivial and that chromosome abnormalities can evolve, especially after therapy, it is relevant to ask whether identifying the key causes of HGC can provide comparable prognostic information to measuring GC itself. Therefore, our hypothesis was that the inclusion of DNA methylation subtype and TL, with GC, into clinical models of patient outcome would refine and improve the detection of patients destined to exhibit aggressive or progressive disease, with inferior overall survival. Our study was the first to evaluate the clinical relevance and independent prognostic significance of GC, TL, IGHV status and methylation epitypes in 495 untreated patients with CLL in the UK (immuno)-chemotherapy trials. We demonstrated that, in addition to GC, epigenetic information and TL data can help identify additional patients with poor survival.

## Methods

### Patient cohort

Our cohort comprised a total of 495 untreated patients with CLL enroled in three randomized clinical trials; the UK LRF CLL4 trial [37] (NCT00004218, *n* = 251); UKCRN ADMIRE [38] (‘ADM’, ID6897, *n* = 122); UKCRN ARCTIC trial (‘ARC’, ID7136, *n* = 122) [39] (Table S1). All patients were diagnosed using established morphological and immunophenotypic criteria. Informed consent was obtained from all participants in accordance with the Declaration of Helsinki and the study was approved by the Somerset Regional Ethics Committee. The assessment of established biomarkers including FISH, CD38, ZAP70 and IGHV mutational status was performed at randomization as previously described [37–39]. The distribution of clinico-biological features was consistent across cohorts (Table 1).

**Table 1:** Baseline clinco-biological features of the ARCTIC, ADMIRE and CLL4 trial patients.

Variable	ARCTIC & ADMIRE	CLL4	Concordance
	*N* (%)	*N* (%)	*P*-value *
Total number of patients	244	251	
Age, median years (range)	63 (36–80)	64 (42–86)	0.057
Male	183 (75%)	184 (73.3%)	0.67
Female	61 (25%)	67 (26.7%)	
Binet Stage A	35 (14.4%)	64 (25.5%)	<0.01
Binet Stage B	127 (52%)	110 (43.8%)	
Binet Stage C	82 (33.6%)	77 (30.7%)	
IGHV Unmutated	129/218 (59.2%)	141/221 (63.8%)	0.32
IGHV Mutated	89/218 (40.8%)	80/221 (36.2%)	
*TP53* Wildtype	217 (88.9%)	227 (90.4%)	0.58
*TP53* mutated	16 (6.6%)	13 (5.2%)	
*TP53* deleted	2 (0.8)	2 (0.8%)	
TP53 biallelic loss	9 (3.7)	9 (3.6%)	
*TP53* Dysfunction	27 (11.1%)	24 (9.6%)	
*ATM* Wildtype	177 (72.6%)	193 (76.9%)	<0.05
*ATM* Deleted	30 (12.3%)	41 (16.3%)	
ATM Mutated	24 (9.8%)	12 (4.8%)	
*ATM* biallelic loss	13 (5.3%)	5 (2%)	
*BIRC3* Wildtype	201 (82.4%)	203 (80.9%)	0.62
*BIRC3* Deleted	27 (11.1%)	36 (14.3%)	
*BIRC3* Mutated	9 (3.7%)	7 (2.8%)	
*BIRC3* biallelic loss	7 (2.9%)	5 (2%)	
Trisomy 12 Wildtype	207 (84.8%)	226 (90%)	0.08
Trisomy 12	37 (15.2%)	25 (10%)	
13q Wildtype	115 (47.1%)	145 (57.8%)	0.05
13q Deletion	104 (42.6%)	81 (32.3%)	
13q biallelic deletion	25 (10.2%)	25 (10%)	
*SF3B1* Wildtype	193 (79.1%)	191 (76.1%)	0.71
*SF3B1* Mutated	51 (20.9%)	60 (23.9%)	
*NOTCH1* Wildtype	209 (85.7%)	212 (84.5%)	0.71
*NOTCH1* Mutated	35 (14.3%)	39 (15.5%)	
n-CLL epitype	103/220 (46.8%)	115/226 (50.9%)	<0.01
i-CLL epitype	61/220 (27.7%)	83/226 (36.7%)	
m-CLL epitype	56/220 (25.5%)	28/226 (12.4%)	
Short telomere length	56 (23%)	137 (54.6%)	<0.01
Intermediate telomere length	62 (25.4%)	61 (24.3%)	
Long telomere length	126 (51.6%)	53 (21.1%)	
Telomere Length, median kb (range)	3.63 (1.13–7.68)	2.88 (1.93–10.24)	<0.01
CNA count, median (range)	2 (0–26)	2 (0–23)	0.01
Mutation Count 0	95 (38.9%)	116 (46.2%)	0.42
Mutation Count 1	97 (39.8%)	87 (34.7%)	
Mutation Count 2	35 (14.3%)	34 (13.5%)	
Mutation Count ≥3	17 (7%)	14 (5.6%)	
Low Genomic Complexity	156 (63.9%)	178 (70.9%)	0.11
Intermediate Genomic Complexity	57 (23.4%)	40 (15.9%)	
High Genomic Complexity	31 (12.7%)	33 (13.1%)	

* Chi squared was used to test the concordance between categorical variables and a Wilcoxon rank test was used for continuous variables. CNA data are generated from genomic techniques, not FISH.

### Targeted sequencing

Mutational data previously generated using an Illumina TruSeq Custom Amplicon panel was available for 454 patients (CLL4 *n* = 228 [40] and ARC/ADM *n* = 226 [41]). An additional 41 patients (CLL4 *n* = 23, ARC/ADM *n* = 18) were analysed using a bespoke Agilent Sureselect XT HS2 Targeted Enrichment System, targeting 63 genes selected for their clinical relevance in B-cell malignancies, according to manufacturer’s protocols. Mutations were identified using custom pipelines and filtered as previously reported [40] (Supplementary Methods). Consensus mutational data was available for 9 recurrently mutated CLL genes (Table S2).

### Copy number profiling

Previously published copy number alteration (CNA) data derived from Affymetrix SNP 6.0 (CLL4, *n* = 108) [42, 43] and the HumanOmni2.5-8.0 SNP (ARC/ADM, *n* = 212) [41] were available. An additional 32 ARC/ADM patients were profiled as previously described [35], using the Illumina Infinium Human Methylation 450 BeadChip (Illumina, Hayward, CA, USA), according to manufacturer’s instructions, at the Genomics and Proteomics Core Facility of the DKFZ (Heidelberg, Germany). Data processing was performed using RnBeads v2.93 (RRID:SCR_010958) and Conumee [44] (Supplementary Methods). The remaining 143 CLL4 patients were profiled using shallow WGS, using the Agilent SureSelect QXT system according to manufacturer’s recommendations as previously described [40, 45]. The data was analysed using custom pipelines (Supplementary Methods).

After passing the inclusion criteria for individual technologies (Supplementary Methods), all CNA were manually curated by two independent experienced researchers, with 1295 CNA, ranging from 7Kb-155MB passing inspection. A high concordance for calling del17p, del11q, Tri12 and del13q was found between the four genomic technologies and FISH data (percentage agreement; 71-99%) (Table S3). Based on CNA burden, including aneuploidy, patients were classified as having Low Genomic Complexity (LGC, 0–2 CNAs, *n* = 334/67%), Intermediate GC (IGC, 3–4 CNAs, *n* = 97/20%) or High GC (HGC, ≥5 CNAs, *n* = 64/13%). CNA identified within known regions of importance (2p, 4p, 6q, 11q, 13q, 14q and 17p) were included in all analyses, regardless of size, while a cutoff of ≥5 Mb was applied for other CNAs, as previously reported [46]. Copy-number neutral loss of heterozygosity and biallelic losses, were excluded and counted as two CNAs, respectively. CNAs within ≤5 Mb of each other and putative chromothripsis events were counted as a single event.

No significant difference was observed in the proportion of each GC group in the CLL4 and ARC/ADM cohorts (Fig. S1). The prevalence of CNAs across the four platforms was also highly concordant, except for a modest reduction in the CNAs detected by sWGS (Fig. S2), due to more cautious CNA definition consequent on higher, but acceptable levels of background noise. To emphasize this point, the sWGS data demonstrated a high concordance with FISH for del17p (98%), del11q (91%), Tri12 (95%) and del13q (80%) (Table S3).

### Telomere length analysis

Telomere length data was generated using Single Telomere Length Analysis (STELA) (ARC/ADM *n* = 232) and monochrome multiplex PCR (MMQPCR) (CLL4 *n* = 180) as previously described [24, 26]. For 80 CLL4 patients analysed with both MMQPCR and STELA, a correlation between technologies was observed (Kendall’s *τ* = 0.657). Additional profiling, using MMQ-PCR, was performed on 71 CLL4 and 12 ARC/ADM patients. Statistical analyses were performed on the combined TL dataset (*n* = 495), as previously reported [24].

### DNA methylation analysis

Previously published DNA methylation data was available on 446 patients (CLL4 = 226, ARC/ADM = 220), derived from pyrosequencing [35]. Patients were classified as naive B-cell–like CLL (n-CLL), memory B-cell–like CLL (m-CLL) and intermediate CLL (i-CLL), using established criteria [35, 47].

### Statistical analysis

Clinico-biological associations were evaluated using the Wilcoxon rank-sum test for continuous variables and Fisher’s exact or chi-square tests for categorical variables, as appropriate based on data distribution and sample size. Statistical significance was defined as *p* < 0.05, with Benjamini–Hochberg correction applied for multiple comparisons. Overall survival (OS) was measured in years from date of randomization to date of death from any cause or date of last follow-up and progression-free survival (PFS) was measured in years from date of randomization to progression (relapse requiring further treatment), date of death or date of last follow-up. CLL4 survival data (*n* = 251) for PFS and OS came from the final updates in 2010 (10 years follow-up) and 2016 (17 years follow-up), respectively (median PFS = 2.4 years, median OS = 6 years). ARC/ADM PFS and OS data (*n* = 244) came from the 2022 update, after 9 years follow-up (median PFS = 4.72 years, median OS = 6.44 years). Kaplan-Meier curves (log-rank test), Cox proportional hazards models and Multivariate Cox Proportional Hazard models using stepwise backwards elimination were used to investigate PFS and OS. Due to the different treatment modalities and follow-up periods for ARC/ADM and CLL4, survival analysis was performed separately for each cohort. Analyses were performed in SPSS (v23) and R(v4.3.0). Code for statistical analysis is available on request.

## Results

### Cohort overview

**Fig. 1:**
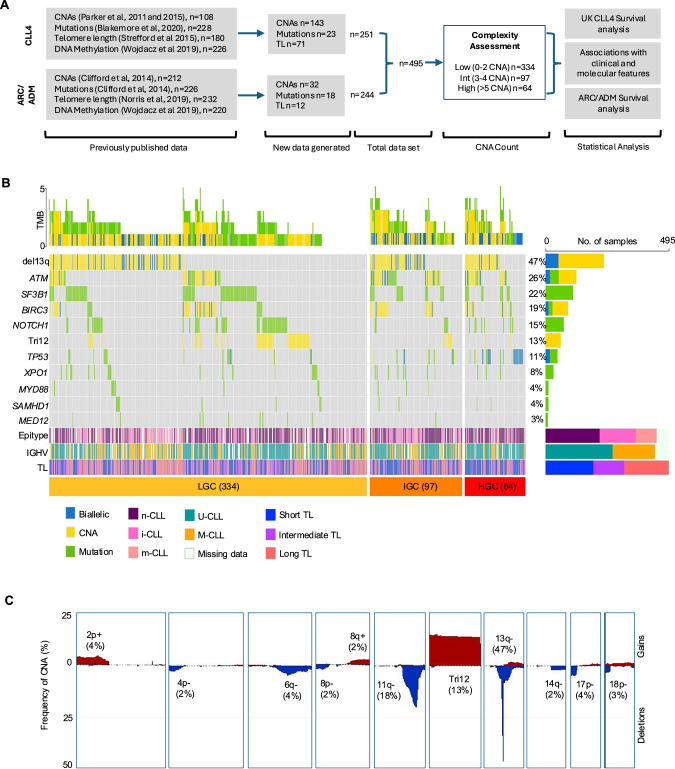
The genomic landscape of CLL. **A** Consort diagram showing the experimental workflow and key datasets in our study. **B** Waterfall plot, segmented by genomic complexity classification (LGC, IGC and HGC), showing the frequency of mutations in the nine profiled genes (green bars) and most common CNA (yellow bars) in the total CLL cohort (*n* = 495). Biallelic loss (deletion + mutation, or biallelic deletion = blue bars). Horizontal bars below show the distribution of epitype groups, IGHV usage and telomere length (TL) for each patient, across the GC subgroups. **C** Frequency plot showing the percentage of patients with recurrent copy-number gains (red) and losses (blue) in 495 CLL patients.

The overview of our cohort design and methodological approaches is depicted in Fig. 1A. Baseline clinico-biological characteristics of the 495 patients with CLL are shown in Table 1. Of these, 367 (74%) were male and 128 (26%) were female, with a median age of diagnosis of 64 years (range: 36–86 years). IGHV mutational status was available for 439 patients, of whom 39% (*n* = 169) had mutated IGHV (M-CLL) and 60% (*n* = 270) had unmutated IGHV (U-CLL) (Fig. 1B). Telomere length ranged from 1.13–10.24Kb (median 3.21 kb), with patients classified as TL-Short (TL-S, <2.92 kb, *n* = 193), TL-Intermediate (TL-I, 2.92–3.57 kb, *n* = 123) or TL-Long (TL-L, >3.57 kb, *n* = 179) using established cut-offs [24] (Fig. 1B). Additionally, methylation-based classification, according to published criteria [47], assigned patients to; n-CLL (*n* = 218, 49%), i-CLL (*n* = 144, 32%) or m-CLL (*n* = 84, 19%) subgroup (Fig. 1B).

In our cohort, 426 variants were identified in 284 patients across the nine consensus genes (*ATM, SF3B1, NOTCH1, TP53, BIRC3, XPO1, MYD88, SAMHD1* and *MED12*), with a median of 0.86 (range: 0–6) mutations/sample. Mutations were detected in all genes, with the highest frequencies identified in *SF3B1* (22%, *n* = 111), *NOTCH1* (15%, *n* = 74), *ATM* (11%, *n* = 54), *TP53* (9%, *n* = 47) and *BIRC3* (6%, *n* = 28) (Fig. 1B and Table S4).

Copy number analysis identified 1297 CNAs in 452/495 patients (mean: 2.62, range: 0–23), including 65 aneuploidy events (Tri12, *n* = 62). Established CLL-associated CNAs were identified at expected frequencies: del17p in 4.4%, del11q in 18%, del13q in 47.4%, tri12 in 12.5% (Fig. 1B, C). Additional recurrent CNA (present in >2% of the cohort) and minimally deleted or enhanced regions were defined on chromosomes 2p, 4p, 6q, 8 and 14 (Fig. 1C and Table S5).

### Genomic complexity associates with clinico-biological features

**Fig. 2:**
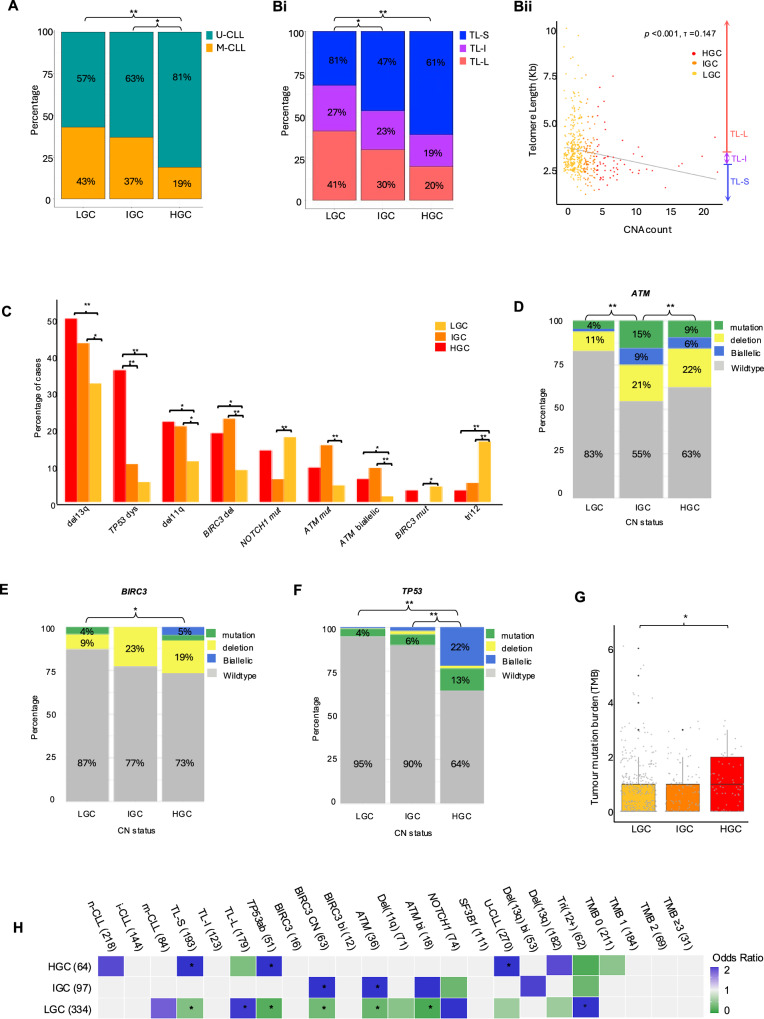
Genomic complexity associates with clinico-biological features. **A** Stacked bar graph showing the proportion of U-CLL (teal) and M-CLL (orange) patients across the 3 GC subgroups. **Bi** Stacked bar graph showing the proportion of patients classified as having TL-S (blue), TL-I (purple) and TL-L (pink) across the 3 GC subgroups. **Bii** Scatterplot showing the significant negative correlation (*p* < 0.001) between telomere length and CNA count. The linear regression model is depicted by the grey line. Points are coloured to indicate GC subgroup (HGC =red, IGC=orange, LGC=yellow). **C** Frequency bar chart showing the features that are significantly associated with HGC, IGC and LGC (red, orange and yellow, respectively). Significant enrichment of features was determined using a Fishers Exact test or Chi-Squared test. Stacked bar graphs showing the proportion of mutations (green), copy number losses (yellow) biallelic losses (blue) and wildtypes (grey) in the *ATM* (**D**), *BIRC3* (**E**) and *TP53* (**F**) genes across the 3 GC subgroups. *P*-values indicate a significant enrichment of gene dysfunction, encompassing deletions, mutations, or both combined. **G** Box and whisker plots showing the increased tumour mutation burden (TMB) in HGC patients compared to LGC and IGC. In all plots * indicates *p* < 0.05, ** indicates *p* < 0.001. **H** Association plot of odds ratios showing features that co-occur with GC subgroups, or are mutually exclusive (blue and green squares, respectively) (*p* ≤ 0.05, corrected *p*-values calculated using Benjamini-Hochberg FDR (*p* ≤ 0.05 *).

First, we analysed the relationship between genomic complexity (GC) subgroups and key clinico-biological features of CLL. HGC was more prevalent in U-CLL (81%, *n* = 47, *p* < 0.001) (Fig. 2A), with significantly higher CNA counts in U-CLL (mean: 2.86) compared to M-CLL (mean: 2.01, *p* < 0.001). TL varied significantly across GC groups, with TL-L and TL-S predominantly occurring in LGC (41%) and HGC patients (61%), respectively (*p* < 0.05) (Fig. 2Bi). As a continuous variable, TL negatively correlated with CNA count (Kendall’s *τ* = –0.147, *p* < 0.001) (Fig. 2Bii).

GC was associated with recurrent chromosomal abnormalities; Monoallelic deletions of chromosome 13q were significantly enriched in HGC (*n* = 32, 50%) compared to LCG (*n* = 108, 32%, *p* < 0.01) (Fig. 2C). As 13q deletion size has previously been linked to GC, we further classified our deletions into Class I (<2 Mb, *n* = 86) and Class II (>2 Mb, *n* = 170) deletions [42], with Class II deletions significantly enriched in HGC (45%) and IGC (49%) (*p* < 0.05). Trisomy 12 was more prevalent in LGC (*n* = 55, 16.5%) versus IGC (*n* = 5, 5.2%) and HGC groups (*n* = 2, 3.1%) (*p* < 0.01) and frequently co-occurred with *NOTCH1* mutations in 24/62 cases, with 22 (92%) occurring in LGC (Fig. 2C).

Deletions of 11q, encompassing the *ATM* gene, were detected in 71 patients and were enriched in HGC and IGC (*n* = 14, 22% and *n* = 20, 21%, respectively). Additionally, 54 patients harboured either a mutation (*n* = 36), or a deletion and a mutation of *ATM* (*n* = 18), both of which were significantly enriched in IGC (*p* < 0.001) (Fig. 2D). *BIRC3* deletions (*n* = 63) did not occur independently of *ATM* loss and were also significantly associated with HGC and IGC cases (*n* = 18 and *n* = 22, respectively, *p* < 0.001), with no significant associations recorded between GC groups and biallelic *BIRC3* inactivation (*n* = 12) (Fig. 2E).

*TP53* aberration, defined as copy number loss, mutation or both, was observed in 51 patients, with 23 (36%) classified as HGC (36%) (*p* < 0.001) (Fig. 2C). Notably, 14 of the 18 cases (78%) with biallelic *TP53* loss were HGC (Figs. 1B and 2F). A modest positive correlation was observed between Tumour Mutation Burden (TMB) and CNA count (Kendall’s *τ* = 0.26, *p* = 0.042), with a higher mean mutation count in the HGC compared to LGC (mean= 1.08 vs 0.83, respectively, *p* < 0.05) (Fig. 2G). *NOTCH1* (*n* = 74) and *BIRC3* mutations (*n* = 16) were more frequently observed in LGC (17 and 8%, respectively) (Fig. 2E–H) and whilst *SF3B1* mutations were not overrepresented in any complexity subgroup, 70% of *SF3B1* mutations (*n* = 79) were found in LGC patients. While GC was not significantly associated with methylation-based subgroups, 62% (*n* = 38) of HGC patients belonged to the n-CLL subgroup (Figs. 2H and S3).

### The clinical implications of high genomic complexity

**Fig. 3:**
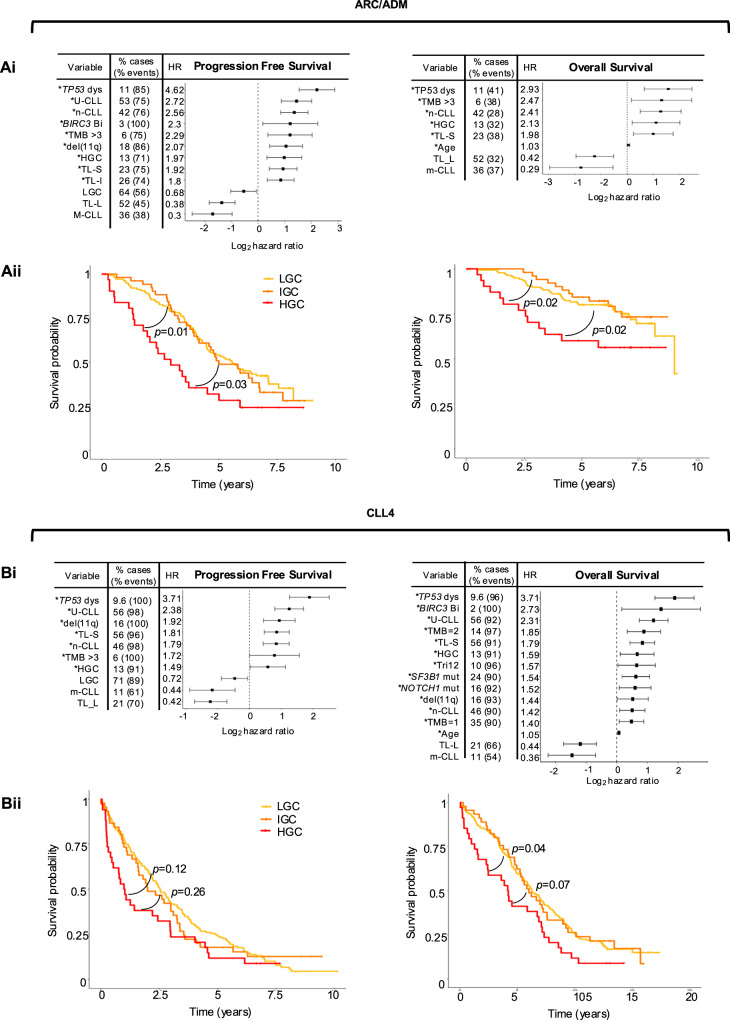
Clinical implications of GC in univariate analysis. **Ai**-**Bi** Forest plots showing the hazard ratios for variables significant (*p* < 0.05) for Progression Free Survival (PFS) and overall survival (OS) in univariate cox regression analysis, for ARC/ADM (**Ai**) and CLL4 (**Aii**). The proportion of cases positive for each variable and the proportion of events in those cases is shown. * indicates poor risk variables included in the multivariate model (MV). Kaplan–Meyer plots showing the shorter PFS and OS in HGC cases in ARC/ADM (**Aii**) and CLL4 (**Bii**).

Due to the different treatment modalities and follow-up periods for ARC/ADM and CLL4 (ARC/ADM; 9 years, CLL4 PFS; 10 years, OS; 17 years) survival analysis was performed separately for each cohort. In both cohorts, HGC was significantly associated with a shorter median PFS and OS compared to LGC (Table S6), as well as significantly lower 5-year survival rates (ARC/ADM; 51% vs 29%, CLL4; 61% vs 42%) (*p* < 0.01). Univariate Cox regression analysis of 13 clinical and molecular features was performed to evaluate their prognostic significance for PFS and OS (Tables S7–8), with significant associations displayed in Fig. 3Ai, Bi.

In both cohorts, patients with HGC showed an increase in the risk of progression compared to those with no HGC (PFS: ARC/ADM: HR = 1.97, *p* = 0.002; CLL4: HR = 1.49, *p* = 0.04. OS:ARC/ADM: HR = 2.13, *p* = 0.015; CLL4: HR = 1.59, *p* = 0.018). Additionally, several established high-risk indicators for PFS were identified in both cohorts, including *TP53* aberration, n-CLL classification, short or intermediate TL, U-CLL and del11q. TMB > 3 mutations also emerged as a significant predictor of reduced PFS in ARC/ADM and CLL4 (ARC/ADM: HR = 2.29, *p* = 0.003; CLL4: HR = 1.72, *p* = 0.046). Reduced OS in the ARC/ADM cohort was predicted by age, short TL, *TP53* aberration, n-CLL classification and TMB > 3 (Fig. 3Ai). In the CLL4 cohort, in addition to these variables, a further seven features were associated with reduced OS, including *NOTCH1* and *SF3B1* mutations (Fig. 3Bi). Additionally, Kaplan-Meier analysis of HGC patients versus IGC and LGC patients, confirmed reduced PFS in ARC/ADM and reduced OS in both ARC/ADM and CLL4 (Fig. 3Aii, Bii).

**Fig. 4:**
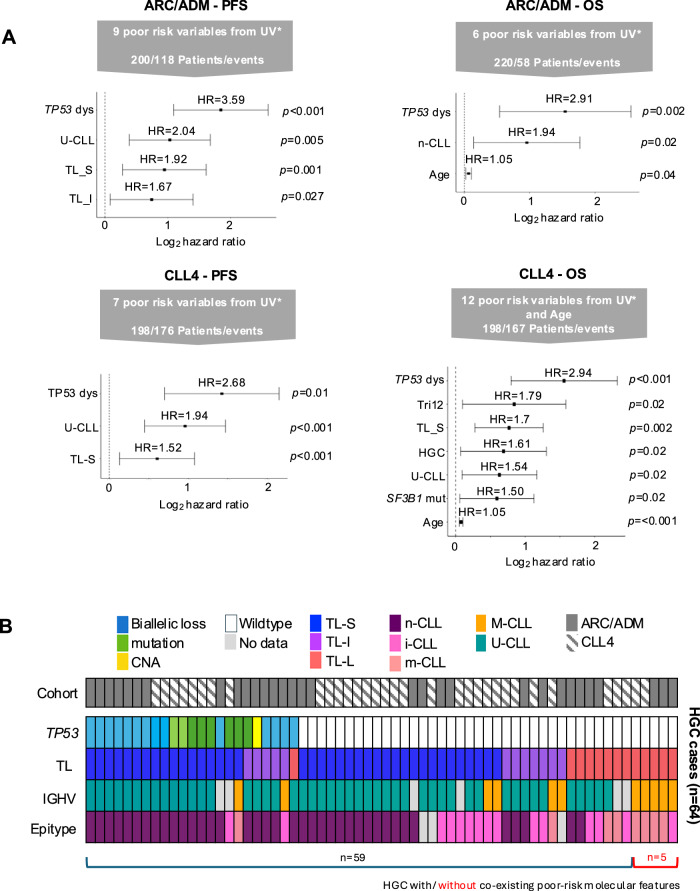
Clinical implications of GC in multivariate modelling. **A** Forest plots showing the hazard ratios for variables significant (*p* < 0.05) for Progression Free Survival (PFS) and overall survival (OS) in multivariate (MV) analysis, for ARC/ADM and CLL4. Grey arrows describe the number of poor risk variables from univariate (UV) analysis and the number of patients and events included in each MV model. Forest plots show the significant independent predictors of outcome in each MV model. **B** Plot showing the distribution of high-risk features in the 64 HGC cases.

Given the co-occurrence of HGC with other high-risk biological features, we next estimated the adjusted impact of HGC on PFS and OS by controlling for these confounders using multivariate Cox proportional hazard analysis with stepwise backward selection. We incorporated high-risk variables, identified through univariate cox regression analysis (Fig. 3Ai, Bi). For PFS, in both ARC/ADM (9 variables tested, 200 patients/118 events) and CLL4 (7 variables tested, 198 patients/176 events), HGC was not a significant independent prognostic marker in the final model (Fig. 4A). Instead, the models included *TP53* aberration (ARC/ADM: HR = 3.59, *p* < 0.001; CLL4: HR = 2.68, *p* = 0.01), unmutated IGHV (ARC/ADM: HR = 2.04, *p* = 0.001; CLL4: HR = 1.94, *p* < 0.01) and TL-S (ARC/ADM: HR = 1.92, *p* = 0.005; CLL4: HR = 1.52, *p* < 0.01) (Fig. 4A). In the ARC/ADM cohort, a model incorporating six high risk variables (220 patients/58 events), identified TP53 aberration (HR = 2.91, *p* = 0.002), n-CLL (HR = 1.94, *p* = 0.02) and age (HR = 1.05, *p* = 0.004) as independent prognostic markers of shorter OS (Fig. 4A). In the CLL4 cohort, a multivariate model for OS, which included 12 poor risk variables and age, (198 patients/167 events), resulted in a final model with seven independent prognostic markers of shorter OS. These included HGC (HR = 1.61, *p* = 0.02), *TP53* aberration (HR = 2.94, *p* < 0.001), Tri12 (HR = 1.79, *p* = 0.002), TL-S (HR = 1.7, *p* = 0.002), U-CLL (HR = 1.54, *p* = 0.02), *SF3B1* mutations (HR = 1.5, *p* = 0.02) and age (HR = 1.05, *p* < 0.001) (Fig. 4A).

Multivariate modelling did not consistently identify HGC as an independent prognostic marker of PFS or OS, suggesting that it may act as a surrogate for other high-risk features, which may underly the observed complexity. Focusing on variables highlighted in our multivariate analysis, we examined the 64 HGC cases across our combined cohorts. Of these, 23 patients exhibited *TP53* aberration, of which 14 also showed co-occurrence of U-CLL, TL-S and n-CLL; 5 had U-CLL and n-CLL; and 3 had TL-S. Among the remaining 41 *TP53* wild-type HGC cases, all but five (7.8%) harboured at least one additional high-risk feature: TL-S (*n* = 18), U-CLL (*n* = 26), or n-CLL (*n* = 17) (Fig. 4B). Taken together, association and survival analyses show that while HGC is linked to poor survival in univariate models, its prognostic value is attenuated in multivariate analysis. Features such as *TP53* aberration, unmutated IGHV, short telomere length and methylation subtype are strongly associated with HGC and independently predict outcome, suggesting that these biomarkers, individually or in combination, may more precisely define this patient subgroup.

## Discussion

This comprehensive (epi)genomic analysis of GC in a large cohort of 495 clinical trial patients underscores the intricate interplay between GC and other high-risk biological features, as well as their collective impact on clinical outcomes. We confirm and extend previous findings by elucidating the relationship between GC and other prognostically relevant molecular characteristics, such as the enrichment of *TP53* lesions and U-CLL in HGC cases [5–7, 10], along with an increased prevalence of trisomy 12 in LGC cases [2].

Notably, our work is the first to include contemporary cell-of-origin measures to identify n-CLL cases and to assess TL, which is closely linked to cellular proliferation. We hypothesized that these measures would better define poor-risk patients not fully captured by traditional GC metrics. Our findings support this, as TL and n-CLL independently identified poor-risk patients, with 92.2% of HGC cases showing *TP53* aberrations, U-CLL, short TL, or being classified as n-CLL. While current clinical paradigms focus on GC, *TP53* and U-CLL for prognostic purposes, our data suggest future research should emphasize DNA methylation signatures and telomere attrition for identifying patients at risk for aggressive disease.

Our work confirms the link between *TP53* aberration, TL shortening and HGC in CLL. *TP53* aberration impairs the DNA damage response, allowing genomic lesions to bypass checkpoints and continue dividing, leading to chromosomal instability [19, 23]. *ATM* deletions were enriched in both HGC and IGC groups, while mutations were more common in IGC, indicating a complex relationship between *ATM* status and GC. Unlike CLL B-cells with *ATM* mutations, those with 11q deletion alone show normal levels of ATM S1981 auto-phosphorylation and p53 S15 phosphorylation after ionising radiation [48]. Our analyses did not show greater GC in bi-allelic *ATM* cases, as might be expected based on functional data, which is in keeping with previous TL data [49], suggesting that other 11q sequences like miR-34 and *H2AFX* may influence GC through altered DNA damage response [50–52]. *BIRC3* mutations were enriched in LGC CLL, reflecting its role as a negative regulator of NF-kB signalling rather than a direct role in DNA damage response [53]. The size of 13q deletions correlates with genomic complexity and disease progression in CLL [54], with large deletions encompassing genes like *RB1* and *RNASEH2B*. Loss of *RNASEH2B* impairs RNase H2 activity, sensitising CLL cells to PARP inhibitors through defective ribonucleotide excision repair [55]. In our study, 66.4% of 13q deletions were large (Class II) [42], suggesting a role in genomic instability and potential sensitivity to PARP inhibition. Mutations in *NOTCH1* and *SF3B1* displayed variable distributions across genomic complexity subgroups and did not independently stratify patient outcomes, with the co-occurrence of *NOTCH1* mutations and trisomy 12 in LGC cases highlighting the heterogeneity of molecular interactions in CLL pathogenesis. A correlation between TL and GC suggests a potential causative link, with telomere shortening possibly preceding GC [18, 56]. Transformation may stabilise TL in B-cells, promoting breakage-fusion-bridge cycles and genomic instability. Alternatively, GC may reflect a more proliferative disease, especially in U-CLL with enhanced BCR signalling [57]. If telomere shortening precedes genomic complexity, telomerase inhibition could disrupt this pathogenic cycle. However, telomerase activity is essential for the long-term proliferative capacity of antigen-specific T cells, particularly CD28⁺ subsets [58], so its inhibition may impair the expansion of B-CLL-specific T cells and potentially compromise immune surveillance [59].

HGC was associated with PFS and OS in both the ARC/ADM and CLL4 cohorts, but it did not consistently retain prognostic independence in multivariate models that included *TP53* abnormalities, TL, n-CLL and IGHV status. This suggests that these co-variates are stronger predictors of outcome and that HGC may primarily act as a surrogate for them, rather than as an independent prognostic marker for poor prognosis. Notably, nearly half of HGC cases harboured *TP53* abnormalities. Biallelic *TP53* loss, an indicator of therapeutic resistance, was especially common in the HGC subgroup, illustrating the role of genomic instability in clonal evolution and resistance mechanisms in CLL. Furthermore, most *TP53* wild-type HGC cases exhibited at least one other high-risk feature (e.g., short-TL, U-CLL, or n-CLL epitype), reinforcing the idea that GC is rarely an isolated phenomenon but rather reflects converging pathways of adverse biology.

This study has several strengths, including: (1) a large, well-characterised cohort with extended clinical follow-up and independent validation in two clinical trials; (2) comprehensive data that build detailed biomarker profiles for each trial participant, including biological variables like TL and DNA methylation epitype, which have not been clinically assessed together with GC until now; (3) the chemotherapy backbone used in these trials, which may provide valuable insights into the association between GC and other biomarkers due to established links between these therapies and genomic instability; and (4) the ongoing and future relevance of chemotherapy with or without rituximab in resource-limited settings [60], where international guidelines continue to recommend screening for *TP53* disruption and IGHV mutation status [61–63], thereby underscoring the need to understand the biological drivers of poor responses to these agents.

However, several limitations must be acknowledged. Firstly, the trials assessed traditional (immuno) chemotherapy regimens that now have limited front-line use in high-income health systems, which may restrict the applicability of our findings in the era of targeted therapies. However, our work accentuates the necessity to investigate the combination of biomarkers in trials of these agents, as sophisticated measures of cell-of-origin, cellular proliferation and genomic instability will likely remain relevant. Indeed, emerging evidence indicates that genomic complexity has independent prognostic significance in some settings, particularly in studies involving venetoclax [64]. Given that TL and epitype are intrinsic, cell-of-origin features and remain stable over time, we anticipate that these factors would offer similar predictive utility in the pre-treatment setting for identifying high-risk patients likely to require therapy. Secondly, a broader gene panel could enhance our understanding of the genomic mechanisms underlying GC, particularly regarding *POT1* mutations, which lead to the accumulation of telomeric and chromosomal abnormalities [65] and the IGLV3-21^R110^ mutation that would help characterise the i-CLL group [34]. Limited data availability prevented a more expansive association analysis of this type. Additionally, our study utilised CNA profiling with limited resolution that cannot identify balanced translocations, which, while rare, represent a proportion of CLL translocations. The broader literature also demonstrates inconsistency in defining and assessing GC, with varying technologies, culturing conditions and thresholds hindering the establishment of a standardised, clinically meaningful metric. Importantly, our study suggests that other biomarkers can effectively identify these poor-risk patients without requiring an international consensus on the most accurate classification of GC.

In conclusion, our findings support a model in which HGC reflects an accumulation of adverse biological features in CLL, including *TP53* aberration, telomere shortening and unmutated IGHV status, particularly among those cases with the most naïve-like epigenetic profiles. Future efforts should focus on longitudinal profiling to map the dynamics of complexity acquisition with telomere attrition in cases characterised at the epigenetic level, along with further validation of our findings in targeted therapy-treated cohorts. Integrating these dimensions into predictive models may enhance risk stratification and enable more personalised management strategies in CLL.

## Supplementary information

Supplementary Methods

## Data Availability

Methylation data are available at Biostudies under accession number E-MTAB-15593. Sequencing data are available at Biostudies under accession numbers E-MTAB-15598 and E-MTAB-15600.
